# Patterns of Temporal Lobe Reaction and Radiation Necrosis after Particle Radiotherapy in Patients with Skull Base Chordoma and Chondrosarcoma—A Single-Center Experience

**DOI:** 10.3390/cancers16040718

**Published:** 2024-02-08

**Authors:** Matthias Mattke, Matteo Ohlinger, Nina Bougatf, Robert Wolf, Thomas Welzel, Falk Roeder, Sabine Gerum, Christoph Fussl, Natalee Annon-Eberharter, Malte Ellerbrock, Oliver Jäkel, Thomas Haberer, Klaus Herfarth, Matthias Uhl, Jürgen Debus, Katharina Seidensaal, Semi Harrabi

**Affiliations:** 1 Department of Radiation Oncology, Paracelsus Medical University, Salzburger Landesklinikum (SALK), 5020 Salzburg, Austria; f.roeder@salk.at (F.R.); s.gerum@salk.at (S.G.); c.fussl@salk.at (C.F.);; 2 Department of Radiation Oncology, Heidelberg University Hospital, 69120 Heidelberg, Germanynina.bougatf@med.uni-heidelberg.de (N.B.); robert.wolf@med.uni-heidelberg.de (R.W.); thomas.welzel@med.uni-heidelberg.de (T.W.); oliver.jaekel@med.uni-heidelberg.de (O.J.); klaus.herfarth@med.uni-heidelberg.de (K.H.); juergen.debus@med.uni-heidelberg.de (J.D.); semi.harrabi@med.uni-heidelberg.de (S.H.); 3 Heidelberg Institute of Radiation Oncology (HIRO), 69120 Heidelberg, Germany; malte.ellerbrock@med.uni-heidelberg.de (M.E.); thomas.haberer@med.uni-heidelberg.de (T.H.); 4 Clinical Cooperation Unit Radiation Oncology, German Cancer Research Center (DKFZ), 69120 Heidelberg, Germany; 5 Heidelberg Ion-Beam Therapy Center (HIT), Department of Radiation Oncology, Heidelberg University Hospital, 69120 Heidelberg, Germany; 6 Institute of Research and Development of Advanced Radiation Technologies (radART), Paracelsus Medical University, 5020 Salzburg, Austria; 7 Division for Medical Physics in Radiation Oncology, German Cancer Research Center (DKFZ), 69120 Heidelberg, Germany; 8 Department of Radiation Oncology, Ludwigshafen Hospital, 67063 Ludwigshafen, Germany; uhlma@klilu.de; 9 National Center for Tumor Diseases (NCT), 69120 Heidelberg, Germany; 10 German Cancer Consortium (DKTK), Partner Site Heidelberg, 69120 Heidelberg, Germany

**Keywords:** chondrosarcoma, chordoma, radiotherapy, heavy ion, carbon ion, skull base, proton, necrosis

## Abstract

**Simple Summary:**

Around 30% of the patients receiving intracranial particle therapy develop radiogenic reactions of the brain. There are a wide range of symptoms, ranging from asymptomatic blood–brain barrier disorders to life-threatening brain necrosis. The aim of our retrospective study is to assess the patterns of occurrence in patients treated for skull base chordoma and chondrosarcoma with protons or carbon ions. Furthermore, we aimed to develop a prognostic model for the prediction of radiation reactions.

**Abstract:**

Background: The current study aims to evaluate the occurrence of temporal lobe reactions and identify possible risk factors for patients who underwent particle therapy of the skull base. Methods: 244 patients treated for skull base chordoma (*n* = 144) or chondrosarcoma (*n* = 100) at the Heidelberg Ion Beam Therapy Center (HIT) using a raster scan technique, were analyzed. Follow-up MRI-scans were matched with the initial planning images. Radiogenic reactions were contoured and analyzed based on volume and dose of treatment. Results: 51 patients with chordoma (35.4%) and 30 patients (30%) with chondrosarcoma experienced at least one temporal lobe reaction within the follow-up period (median 49 months for chondrosarcoma, 62 months for chordoma). Age, irradiated volume, and dose values were significant risk factors for the development of temporal lobe reactions with the highest significance for the value of DMax-7 being defined as the dose maximum in the temporal lobe minus the 7cc with the highest dose (*p* = 0.000000000019; OR 1.087). Conclusion: Temporal lobe reactions are a common side effect after particle therapy of the skull base. We were able to develop a multivariate model, which predicted radiation reactions with a specificity of 99% and a sensitivity of 52.2%.

## 1. Introduction

Chordoma and chondrosarcoma of the skull base are rare malignant bone tumors. Because of the low potential for metastasis, the primary form of treatment is local control [1], whereby function preserving surgery followed by particle radiotherapy is currently regarded as the gold standard. The effectiveness of this approach has been shown in several studies [2,3,4]. Therapy is usually well tolerated, but blood–brain barrier (BBB) disorders and radiation necrosis remain the most common forms of tissue damage following treatment [5,6]. The BBB prevents harmful substances from entering the brain from the bloodstream. This physiological barrier also maintains the special internal environment that the sensitive brain needs to function properly. Disorders in the BBB can cause changes in the homeostasis of the brain with manifold further effects.

Bojaxhiu et al. found radiogenic reactions of the brain in about 28% of the patients after proton therapy for primary brain tumors [7]. BBB disorders and consecutive MRI contrast enhancements in the brain parenchyma may be seen as a preliminary stage of radiation necrosis. These are milder forms of injury that primarily affect the white matter of the brain. As long as clinical outcomes remain stable, biopsy is not recommended, but patients should be routinely monitored for onset of radio-necrosis [8].

Symptoms associated with radiation necrosis vary widely. In severe cases, they present as neurological deterioration, loss of function, or even death [9]. Asymptomatic courses are common as well [10] and are dependent on many, not fully understood factors. Possible explanations may be the choice of radiation modality, the applied dose, or patient-specific variables, e.g., age. Due to this multifactorial genesis, an average incidence is hard to determine. For example, an increased risk for temporal and frontal lobe necrosis has been described when radiating skull base tumors with carbon ions [11]. It has been shown that 80% of radiation necrosis occurs in the first three years after the end of therapy. However, their emergence is variable; there are documented cases a few weeks after the end of the radiotherapy and up to 19 years post treatment [12]. Necrosis usually appears as a single lesion in MRI images, but multiple lesions, and even lesions contralateral to the target volume, have been described. Furthermore, in some patients radiation necrosis occurred in multiple places in the brain in the course of several follow-up examinations [13].

There are various hypotheses related to the pathophysiology of radiation necrosis. The glial hypothesis postulates that radiation directly damages the sensitive oligodendrocytes resulting in a demyelination of nerve tissue [14]. The vascular hypothesis suggests a more indirect effect of radiation on demyelination and ischemic necrosis, which involves injuries to small vessels [15].

Biopsies of the brain show low sensitivity and specificity, and therefore, reliable diagnosis of radiation necrosis is only possible through surgical pathology. As open brain surgery is a major intervention, MRI has become a critical tool for diagnosing brain necrosis. On T1-weighted MRI, a radiation necrosis typically shows a ring-shaped contrast enhancement, while its central necrotic area is hypointense [8,16]. In the T2 sequences, the outer edge shows a lower signal and the central necrotic area is hyperintense [13].

Of particular interest is the reliable differentiation between radiation necrosis and tumor progression, often difficult to distinguish due to their morphological similarities when viewed in MRI images. A misdiagnosis and false therapeutic decisions can result in fatal consequences. For further discernment, radiologists employ several modalities, such as perfusion imaging, T1/T2 matching or positron emission tomography, whereas a combination of different imaging methods shows the greatest sensitivity [17]. Nevertheless, an operation may be indicated in bulky lesions, if lesions are progressive or refractory concerning conservative treatment [12].

Despite BBB disorders and temporal lobe reactions being the most common side effects of radiation to skull base cancers, the literature remains scarce, especially concerning particle therapy. Therefore, we initiated the present analysis. The goals of this study were (1) to describe the incidence of radiogenic brain lesions after particle treatment of skull base chordoma and chondrosarcoma and (2) to develop a model which could be used to predict the risk of radiogenic lesions based on patient and therapy-specific criteria and thus provide information strategies for minimizing these risk factors.

It needs to be mentioned that the prevailing analysis is a subproject within our task force for the further research of radiation necrosis. Further research is being conducted at the moment and will be published in the near future.

## 2. Materials and Methods

Our study was a retrospective analysis of all patients 18 years or older, who were treated for classic and chondroid skull base chordomas or G1–G2 chondrosarcomas at the Heidelberg Ion Beam Therapy Center (HIT) in Heidelberg, Germany, between 2009 and 2014. Two patients who presented with dedifferentiated chordomas were excluded due to their short follow-up period. Detailed information on the patient cohort and the facility has been previously published [3,4]. Prior to 2013 and based on published findings from the Institute for Heavy Ion Research (GSI, Darmstadt, Germany), where no proton therapy was available, HIT patients received carbon ion treatment as standard therapy. With continued research, this practice was changed in 2013 to the worldwide standard of care using protons and carbon ion therapy at HIT was only offered to patients as part of a prospective randomized trial. This explains the longer median follow-up for carbon ions.

Carbon ion doses used in this study were 66 Gy RBE for chordoma and 60 Gy RBE for chondrosarcoma at 3 Gy RBE fraction dose. The proton prescription doses were 74 Gy RBE for chordoma and 70 Gy RBE for chondrosarcoma at 2 Gy RBE fraction dose.

### 2.1. Follow-Up

Patients were scheduled for follow up visits, including MRI of the skull base, every 3 months for the first two years and on an individual basis thereafter. Virtual follow-up examinations with in-house review of outpatient MRI imaging, and telemedicine for symptom assessment, was made possible based on patients’ travel distances and requirements. All data were collected in the central HIRO research database within the authors’ facility, as previously described [18]. If radiogenic reactions or radiation necrosis were found in a patient’s records, the corresponding follow-up MRI was imported into the planning software. Consequently, the temporal lobe reaction/necrosis was contoured and correlated with the initial treatment plan. The dose in the corresponding area, the irradiated volume at defined dose levels as well as the median dose to the temporal lobe were then evaluated.

### 2.2. Differentiation between Radiation Reaction and Radiation Necrosis

**Figure 1:**
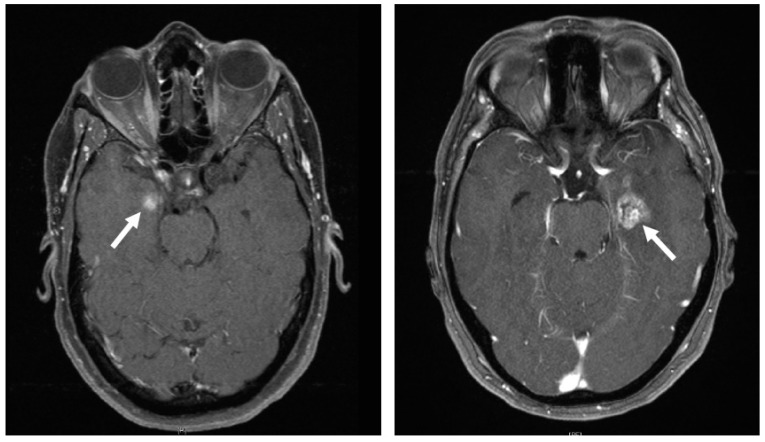
The arrows point to examples for temporal lobe reaction (**left**) and necrosis (**right**).

The differentiation between temporal lobe reactions and necrosis can be challenging, particularly because the former often transitions fluidly into the latter (Figure 1). A board-certified radiologist with extensive experience with skull base tumors assessed all follow-up MRI scans. Distinction between BBB disorders (temporal lobe reactions) and radiation necrosis were made using the most recent MRI with contrast enhanced T1-sequence, T2-sequence, SWI-sequence, previous MRI, development over the course of time, and, if available, other modalities of radiologic examination.

If a patient had a reaction and necrosis, both were evaluated independently.

### 2.3. Statistical Analysis

Within the planning software (Siemens Healthineers, Erlangen, Germany), the actual radiation plan was selected and a detailed dose–volume histogram (DVH) of the cumulative dose was created from the basic and boost for all relevant structures. The corresponding dose–volume values were then imported into Microsoft Excel^®^ (Microsoft Corp., Redmond, WA, USA) for further evaluation. Using the programming language Visual Basic for Applications, we developed a script which extracted values of interest based on their uniform nomenclature and inserted these values into a table. Thus, for both temporal lobes, the minimum, maximum, average, and median doses were collected.

Additionally, dose variables were defined to determine the maximum dose in the total volume of the temporal lobes, of which a defined volume with the highest dose was subtracted. Accordingly, the variables DMax-1, DMax-2, DMax-3, DMax-4, DMax-5, DMax-7, DMax-10, DMax-15, DMax-20, DMax-30, DMax-40, and DMax-50 were defined as highest dose within the volume of the temporal lobe, minus one specific cubic centimeters, two cubic centimeters, etc., with the highest dose.

The volume for temporal lobes irradiated with 10, 20, 30, 40, 50, 60, 70, or 80 (V10–80) Gy (RBE) was determined, and likewise, the minimum, maximum, mean, and median dose during incidences of radiation necrosis, temporal lobe reactions, and contrast enhancements were extracted. Three chordoma patients and one chondrosarcoma patient were excluded from these evaluations because their treatment plan was based on two different planning CT scans, and thus, no reliable calculation of the cumulative dose was possible using their base and boost plans.

A descriptive evaluation of all lesions was performed using IBM SPSS Statistics^®^ (IBM Corp., Armonk, NY, USA). A stratified analysis of the tumor was performed in order to detect variances in the occurrence of radiation necrosis between chordoma and chondrosarcoma patients. To increase the number of cases and thus the statistical reliability, the occurrence of temporal reactions as well as necrosis was included in the model. For this purpose, a univariable analysis was performed to identify significant prognostic parameters that may promote the occurrence of lesions in the temporal lobe. The variables incorporated into this analysis included, the age of the patient at the start of therapy, gender, radiation modality, as well as the dose and volume variables.

Dose and volume variables were collected for each temporal lobe, so that in 244 patients 488 temporal lobes were evaluated. However, the mean values of the total temporal lobes were not compared individually, under the presumption that there may be patient-specific factors, not yet identified. In addition, variables such as age, gender, and radiation modality are patient-specific, not particular to the temporal lobe.

Due to the structure of the data, a generalized, mixed-effects model for binary outcomes was selected for further evaluation. This model takes into account random effects. The occurrence of a lesion in the temporal lobe was defined as a dependent variable, the identity of the patients as a random effect, and the variable to be analyzed as an independent variable.

Based on the results of the univariable analysis, significant variables were selected to create a multivariate forecast model. The generalized, mixed-effects model for binary outcomes was used again. Diagrams and graphs were created using IBM SPSS Statistics^®^. An alpha level of <0.05 was considered significant throughout the evaluation.

The differences in proton versus carbon ion fractionation were also considered. A separate analysis of the carbon ion cases adjusted for EqD2 was performed with no difference in the results.

## 3. Results

### 3.1. Lesions in Chordoma Patients

Temporal lobe reactions were found in 31 patients (21.5%) with a total of 58 lesions, while temporal lobe necrosis was found in 20 patients (13.9%) with a total of 23 lesions. As there was an overlap between the two groups, the total number of patients with at least one lesion was 44 (30.6%), with 24 men and 20 women. A total of 11 of these patients received proton therapy and 33 received carbon ion therapy. The rate of patients with a reaction after radiotherapy in the two treatment groups were 31.4% (11/35) for protons and 30.3% (33/109) for carbon ions.

Of the 288 temporal lobes evaluated in 144 patients, 63 (23%) had at least one lesion.

The median time until first manifestation of the reactions was 20 months (range, 6–73); the median age at the time of manifestation was 56 years (range, 31–80).

The median dose in the contoured volumes of temporal lobe reactions was 63.82 Gy (RBE) (range, 50.56–68.14; mean dose 62.85 Gy (RBE)); the median dose in the contoured volumes of necrosis was 63.97 Gy (RBE) (range, 36.86–70.96; mean dose 61.69 Gy (RBE)). There was no significant difference in the median doses (*p* = 0.9). Further investigation showed significantly lower minimal doses and significantly higher maximal doses in temporal lobe necrosis (*p* < 0.01).

### 3.2. Lesions in Chondrosarcoma Patients

Temporal lobe reactions were found in 18 patients (18%) with a total of 21 lesions, while temporal lobe necrosis was found in 12 patients (12%) with a total of 12 lesions. As in the chordoma group, there was an overlap between patients with reaction and with necrosis; there were a total of 25 (25%) patients with temporal lobe lesions, of which 6 were treated with protons, and 19 with carbon ions. The corresponding rates were 27.3% (6/22) and 24.4% (19/78), respectively. Of the 200 temporal lobes evaluated, stemming from 100 patients, 27 lobes (13.5%) had at least one lesion.

The median time until first manifestation of lesions was 14 months (range, 2–53 months) and the median age at the time of manifestation was 53 years (range, 26–77 years). The median dose in the contoured volumes of temporal lobe reactions was 58.76 Gy (RBE) (range, 48.52–62.00; mean dose 58.04 Gy (RBE)); the median dose in the contoured volumes of necrosis was 61.76 Gy (RBE) (range, 38.76–67.93; mean dose 59.94 Gy (RBE)). The differences in all doses (median, minimal, maximal) were not significant with *p*-values between 0.11 and 0.66.

### 3.3. Univariable Analysis of Risk Factors for the Development of Temporal Lobe Lesions

Several patient- and temporal lobe-specific factors were identified as possible risk factors in the development of lesions in the temporal lobe. These were age at the start of therapy, gender, radiation modality, and a number of temporal lobe specific variables derived from the dose–volume histogram. As it was unclear which variable has the highest predictive power, several variables were defined.

The univariable analysis shown in Table 1 using the generalized binary logistic mixed-effects model helps to identify significant factors. The table mean value of the factors being investigated in the corresponding cohort, as well as the *p*-value showing the probability that the corresponding factor does not have an influence on the development of temporal lobe reactions, is listed. Also recorded is the odds ratio.

For Dmin and V80, no univariable analysis was performed because less than 10% of the corresponding values were not zero; thus, no meaningful statistical analysis could be performed.

As seen in Table 1, the age at the start of radiotherapy had a significant influence on the development of temporal lobe lesions. The average age of the 69 patients who developed a reaction was eight years older than those patients who did not show any temporal lobe reactions. Sex and radiation modality did not have any statistical significance on temporal lobe reactions.

All investigated dose variables had a highly significant influence on the development of lesions. With a mean DMax-7 of 33.09 Gy (RBE) in the cohort without lesions and 49.06 Gy (RBE) in the cohort with lesions, the highest significance level (*p* = 0.000000000019) was seen here. If the dose value is increased by 1 Gy (RBE), the likelihood of developing a lesion increased by the factor of 1.087 (95% confidence interval 1.062–1.114).

Similarly, all investigated volume variables had a significant influence on the development of temporal lobe reactions. V60 as the volume, which was irradiated with at least 60 Gy (RBE), showed a highly significant influence on the development of lesions. In the two cohorts, it was 1.42 cc vs. 4.35 cc with a *p*-value of 0.000000000021. If V60 increased by 1 cc, the likelihood of developing a reaction increased by the factor of 1.395 (95% confidence interval 1.268–1.535).

The analysis of the carbon ion cases adjusted for EqD2 showed similar, highly significant results.

### 3.4. Multivariate Prognostic Model for the Development of Temporal Lobe Reactions

Based on the results of the univariable analysis, age at treatment start and DMax-7 were chosen to be included in the multivariate prognostic model. Age was the only significant patient specific factor, while DMax-7 was the temporal lobe specific factor with the highest level of significance. The inclusion of further variables did not result in an increased accuracy of the model. The results of the prognostic model can be found in Table 2. The model accurately predicted 90.4% of the outcomes with regard to the formation of temporal lobe lesions. The test specificity and sensitivity is 99.0% and 52.2%, respectively.

**Figure 2:**
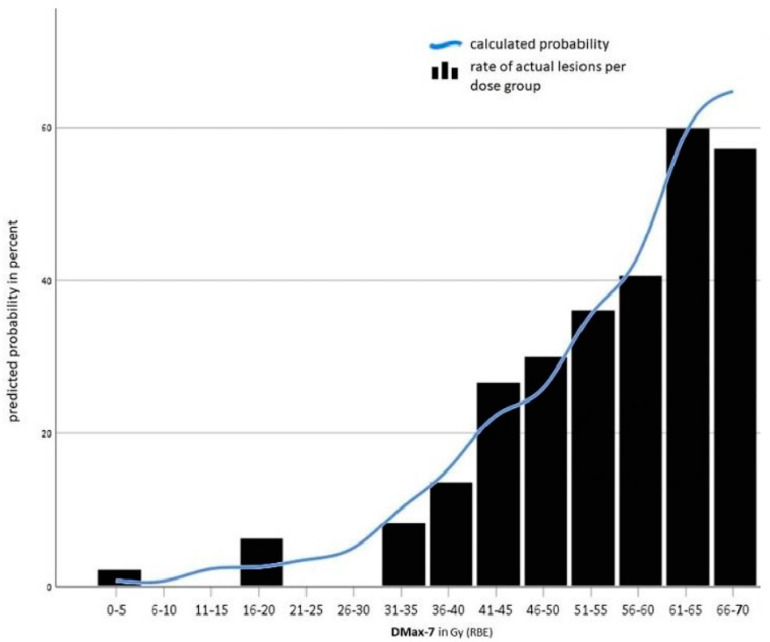
Calculated probability of temporal lobe lesions dependent on DMax-7.

**Figure 3:**
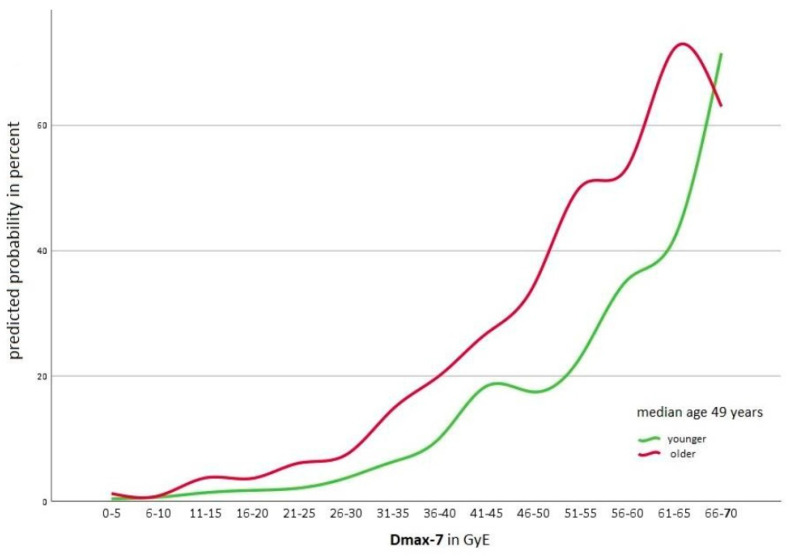
Calculated probability of temporal lobe lesions dependent on DMax-7 stratified for age.

As in the univariable analysis, age and DMax-7 were also significant in the multivariate analysis with a *p*-value < 0.05. Figure 2 shows the probability in all age groups of suffering from a temporal lobe lesion depending on DMax-7 as well as the rate of lesions in the corresponding dose group. As shown in Figure 3, the probability of suffering from a temporal lobe reaction is age-dependent. Except in the highest dose group (66–70 Gy (RBE)), patients older than the median age of 49 had an increased risk of suffering from a temporal lobe reaction.

**Figure 4:**
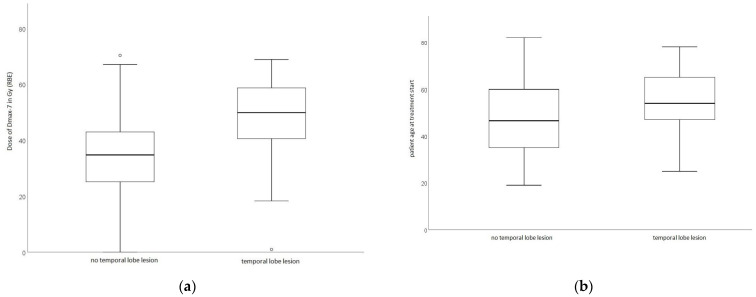
(**a**,**b**): Distribution of DMax-7 and patient age in the groups with and without temporal lobe lesions.

The boxplots in Figure 4a,b show the distributions of DMax-7 and patient age in the groups with and without temporal lobe lesions.

## 4. Discussion

### Temporal Lobe Reactions

Even though temporal lobe reactions are described as the most common tissue damage in the radiotherapy of skull base chordoma [6], published data are scarce. To the best of our knowledge, the present study included the largest cohort evaluated specifically for temporal lobe reactions. Our approach excluded certain volumes with the highest doses (DMax-*) in order to eliminate small to moderately sized dose maxima as possible confounders (point maxima).

A cohort consisting of 59 patients with skull base chordoma and chondrosarcoma, treated with carbon ions in a similar technique as presented in this study, was analyzed by Schlampp et al. at the HIT precursor facility in Darmstadt, Germany in 2011 [19]. There were no overlaps with the patient population in that study and the cohort presented here.

The overall estimate of temporal radiation reactions within the literature is relatively broad (3.3–56.4%) [19,20] and likely based on variations in definitions, imaging techniques, follow-up plan, and patient selection [19,20]. Our resulting rate of 30.6% is in line with the findings published by Lannalfi et al. (39/135; 28.88%) [2] and Mizoe et al. (5/19; 26.31%) [20]. Interestingly, our rate also aligns with that of Bojaxhiu et al., who reported on 171 pediatric patients who were treated with protons for primary brain tumors; 11% of these patients developed a temporal lobe reaction, and 17%, developed radionecrosis [7].

The median time of 20 months until the first presentation of a radiation reaction, observed in this study, is validated by the published literature. Schlampp et al. described a median time of 14 months [19], Miyawaki et al. stated 17 months for protons and 21 months for carbon ions [21], and McDonald et al. stated 21 months [22].

Possible risk factors for the development of a temporal lobe reaction are not yet fully understood. Our study found patient age to be a significant influence, except in the highest dose group (66–70 Gy (RBE)). As this specific dose level only included eight temporal lobes, the results may be unreliable. The literature concerning the influence of patient age is not conclusive. Santoni et al. [23] and Koto et al. [24] were able to show higher rates of reactions in older patients, but the results were not significant. Schlampp et al. [19] observed a significant difference based on age. The risk multiplies by a factor of 1.006–1.096 per year increase in age, which is in line with the results of our study.

A comparison between carbon ion and proton radiation modalities is difficult due to limited data. Miyawaki et al. reported on a higher risk using carbon ions (protons 17%; carbon ions 64%) with comparable doses [21]. No significant differences could be found in the data published by Takagi et al. [25] as well as in the data by Gillmann et al. [26], who compared their proton data to the carbon ions results published by Schlampp et al. [19]. This coincides with the data of the current study, which also showed no significant differences. Notably, differences in the used RBE-models may account for deviating results despite comparable RBE-weighted doses.

With respect to dose variables being predictors for temporal lobe reactions, Su et al. was able to show a significant dependency of radiation necrosis on the Dmax and the D1cc [27], which is in line with the results of the prevailing study. Furthermore, Schlampp et al. [19] were able to show a significant influence of the Dmax v-1 cm^3^ on the emergence of temporal lobe reactions (*p* = 0.001). Dmax v-1 cm^3^ was described as one of the most important predictors for temporal lobe reactions. The same variable showed significant influence within the present study as well.

The current study was able to show that small high-dose areas are more likely to be responsible for temporal lobe reactions than widespread dose coverage. Santoni et al. reported an increase in risk of outcome with a maximum for V30, but the results were not significant. Schlampp et al. did not find any significant results [19]. This may be explained by the chosen threshold of V85, which was rarely attained by most patients. Koto et al. were able to show a higher risk, if the dose was above the median for the V40 and V50 [23].

A higher rate of temporal lobe reactions was seen in chordoma patients than in chondrosarcoma patients (30.3% vs. 25%). This was not surprising seeing that the prescription dose was generally higher in chordoma patients, therefore resulting in a higher delivered dose to the temporal lobes, which in turn is associated with higher risks of toxicity.

A separate analysis comparing the carbon ion cases, adjusted for EqD2, with the proton cases (separately for chordoma and chondrosarcoma), showed no difference in the results. Thus, the different fractionation schemes seem to have no influence within the current cohort.

This study has some limitations. Despite the rather large cohort of patients, data were derived from a single center and were analyzed retrospectively. With its rather low sensitivity, the analyzed metrics are not suitable for being utilized as safety cut-offs in the planning process, e.g., as a metric in planning guidelines. Furthermore, the developed model has only been tested on a single patient cohort. Due to the retrospective character of the study and the lack of detailed information concerning prior surgical approaches, the influence of the surgical extend could not be determined. Lastly, the findings are exploratory and differences in statistical significance concerning the Dmax-*cc metrics are marginal. It may be a unique effect of this dataset, that DMax-7 has the highest statistical significance. External validation will be required before definitive statements regarding reliability can be made.

## 5. Conclusions

The results of this study found correlations between volume variables and radiation reactions in the temporal lobe, especially in the case of high dose values. The combination of age and DMax-7 may be a good predictor for potential temporal lobe reactions and therefore useful in making clinical decisions.

## Figures and Tables

**Table 1:** Univariate analysis of possible prognostic factors for temporal lobe lesions.

Patient Specific Factors	No TL Lesion, *n* = 175	TL Lesion, *n* = 69	*p*-Value	Odds Ratio (95% Confidence Interval)
Age	46.6	54.6	0.00056	1.033 (1.014–1.052)
Sex	49% male 51% female	52% male 48% female	0.97	-
Radiation modality	77% carbon ions 23% protons	75% carbon ions 25% protons	0.647	-
**TL specific factors**	**TL without lesion, *n* = 398**	**TL with lesion, *n* = 90**	***p*-value**	**Odds ratio (95% confidence interval)**
DMin	0.06	0.00003	-	-
DMax	60.00	69.06	0.000010	1.107 (1.058–1.157)
DMean	12.67	17.64	0.00000080	1.090 (1.054–1.127)
Dmedian	7.62	11.23	0.00074	1.044 (1.018–1.070)
DMax-1	50.29	64.14	0.000000063	1.124 (1.078–1.172)
DMax-2	46.12	61.64	0.0000000043	1.111 (1.074–1.151)
DMax-3	42.56	59.15	0.00000000034	1.103 (1.070–1.136)
DMax-4	39.63	56.57	0.000000000066	1.096 (1.067–1.126)
DMax-5	37.13	53.94	0.000000000029	1.091 (1.064–1.119)
DMax-7	33.09	49.08	0.000000000019	1.087 (1.062–1.114)
DMax-10	28.84	42.97	0.000000000067	1.087 (1.061–1.114)
DMax-15	23.75	35.60	0.00000000071	1.087 (1.059–1.116)
DMax-20	19.30	29.86	0.0000000023	1.083 (1.055–1.111)
DMax-30	11.70	19.98	0.000000052	1.071 (1.045–1.097)
DMax-40	6.51	11.48	0.000018	1.059 (1.032–1.088)
DMax-50	3.31	6.52	0.00028	1.063 (1.028–1.098)
V10	29.93	41.47	0.00000096	1.041 (1.025–1.058)
V20	21.66	33.17	0.000000021	1.055 (1.036–1.075)
V30	12.28	22.35	0.00000000014	1.092 (1.063–1.121)
V40	6.12	12.28	0.00000000043	1.130 (1.088–1.174)
V50	3.42	7.87	0.000000000082	1.215 (1.147–1.287)
V60	1.42	4.35	0.000000000021	1.395 (1.268–1.535)
V70	0.18	0.59	0.00118	1.559 (1.193–2.037)
V80	0.0002	0.0008	-	-

**Table 2:** Multivariate prognostic model for the development of temporal lobe lesions.

Portion of accurately predicted temporal lobes with lesions	52.2%
Portion of accurately predicted temporal lobes without lesion	99.0%
Portion of overall accurately predicted temporal lobes	90.4%
*p*-value age	0.00089
Odds ratio age	1.035 (1.014–1.056)
*p*-value DMax-7	0.000000000028
Odds ratio DMax-7	1.089 (1.062–1.116)

## Data Availability

The data that support the findings of this study are available from the corresponding author upon reasonable request.
